# Diagnostic value of striatal ^18^F-FP-DTBZ PET in Parkinson’s disease

**DOI:** 10.3389/fnagi.2022.931015

**Published:** 2022-07-22

**Authors:** Xiu-Lin Liu, Shu-Ying Liu, Olivier Barret, Gilles D. Tamagnan, Hong-Wen Qiao, Tian-Bin Song, Jie Lu, Piu Chan

**Affiliations:** ^1^Department of Neurology, Xuanwu Hospital, Capital Medical University, Beijing, China; ^2^Chinese Institute for Brain Research (CIBR), Beijing, China; ^3^CEA, CNRS, MIRCen, Laboratoire des Maladies Neurodégénératives, Université Paris-Saclay, Fontenay-aux-Roses, France; ^4^Mental Health PET Radioligand Development (MHPRD) Program, Yale University, New Haven, CT, United States; ^5^Department of Radiology and Nuclear Medicine, Xuanwu Hospital, Capital Medical University, Beijing, China; ^6^National Clinical Research Center for Geriatric Diseases, Beijing, China

**Keywords:** 18F-FP-DTBZ, VMAT2, Parkinson’s disease, diagnostic value, positron emission tomography

## Abstract

**Background:**

^18^F-FP-DTBZ has been proven as a biomarker for quantifying the concentration of presynaptic vesicular monoamine transporter 2 (VMAT2). However, its clinical application is still limited.

**Objectives:**

To evaluate the difference in dopaminergic integrity between patients with Parkinson’s disease (PD) and healthy controls (HC) using ^18^F-FP-DTBZ PET *in vivo* and to determine the diagnostic value of standardized uptake value ratios (SUVRs) using the Receiver Operating Characteristic (ROC) curve.

**Methods:**

A total of 34 PD and 31 HC participants were enrolled in the PET/MR derivation cohort, while 89 PD and 18 HC participants were recruited in the PET/CT validation cohort. The Hoehn–Yahr Scale and the third part of the MDS-Unified Parkinson’s Disease Rating Scale (MDSUPDRS-III) were used to evaluate the disease staging and severity. All assessments and PET scanning were performed in drug-off states. The striatum was segmented into five subregions as follows: caudate, anterior dorsal putamen (ADP), anterior ventral putamen (AVP), posterior dorsal putamen (PDP), and posterior ventral putamen (PVP) using automatic pipeline built with the PMOD software (version 4.105). The SUVRs of the targeted subregions were calculated using the bilateral occipital cortex as the reference region.

**Results:**

Regarding the diagnostic value, ROC curve and blind validation showed that the contralateral PDP (SUVR = 3.43) had the best diagnostic accuracy (AUC = 0.973; *P* < 0.05), with a sensitivity of 97.1% (95% CI: 82.9–99.8%), specificity of 100% (95% CI: 86.3–100%), positive predictive value (PPV) of 100% (95% CI: 87.0–100%), negative predictive value (NPV) of 96.9% (95% CI: 82.0–99.8%), and an accuracy of 98.5% for the diagnosis of PD in the derivation cohort. Blind validation of ^18^F-FP-DTBZ PET imaging diagnosis was done using the PET/CT cohort, where participants with a SUVR of the PDP <3.43 were defined as PD. Kappa test showed a consistency of 0.933 (*P* < 0.05) between clinical diagnosis and imaging diagnosis, with a sensitivity of 98.9% (95% CI: 93.0–99.9%), specificity of 94.4% (95% CI: 70.6–99.7%), PPV of 98.9% (95% CI: 93.0–99.9%), NPV of 94.4% (95% CI: 70.6–99.7%), and a diagnostic accuracy of 98.1%.

**Conclusions:**

Our results showed that an SUVR threshold of 3.43 in the PDP could effectively distinguish patients with PD from HC.

## Introduction

Parkinson’s disease (PD), the second most common neurodegenerative disorder (Savica et al., 2016), is currently one of the fastest growing neurological disorders worldwide according to age-standardized prevalence and disability and mortality rates (Dorsey et al., 2018). The cardinal pathophysiology underlying PD is the massive loss of dopaminergic neurons in the substantia nigra (SN), followed by decreased dopaminergic supply in the striatum, which leads to the onset of motor symptoms (Blesa et al., 2022). Resting tremor, rigidity, bradykinesia, and postural instability, recognized as the cornerstone of several clinical diagnostic criteria (Postuma et al., 2015; National Institute for Health and Care Excellence[Nice], 2017; Rogers et al., 2017), are the classic motor features in PD. However, even for experts in movement disorders, the diagnostic accuracy is 79.6% for the initial evaluation and 83.9% after follow-up (Rizzo et al., 2016).

Until recently, the diagnostic procedures of PD were mainly based on clinical symptoms; however, they have gradually developed into a biomarker-supported process (Tolosa et al., 2021). Moreover, neuroimaging is one of the most developed areas in terms of objectivity, veracity, and quantification for the early diagnosis of PD. Positron emission tomography (PET), with emerging and various radioligands targeting the nigrostriatal structures, is credible for quantifying dopaminergic deficit. Dopamine transporter (DAT), vesicular monoamine transporter 2 (VMAT2), and aromatic-amino-acid decarboxylase (AADC) are three key proteins generally targeted for studying the presynaptic dopaminergic system in PD. VMAT2, a specific channel protein taking charge of packing monoamines into synaptic vesicles, has been proven to be less susceptible to compensatory regulation (Kilbourn, 1997; Lee et al., 2000; Nandhagopal et al., 2011) and dopaminergic drug treatments (Borght et al., 1995b; Wilson and Kish, 1996) compared to DAT and AADC. Albeit VMAT2 expressed in all monoaminergic (dopaminergic, serotonergic, norepinephrinergic, or histaminergic) neurons in the CNS (central nervous system) (Scherman et al., 1988), more than 95% of striatal VMAT2 binding sites are dopaminergic axons (Borght et al., 1995a). Additionally, DAT density is 10–20-fold lower than that of VMAT2 in the striatum (Sun et al., 2012). Consequently, tracers targeting at VMAT2 might be more objective for the quantification of presynaptic dopaminergic degeneration. Among them, ^18^F-9-fluoropropyl-dihydrotetrabenazine (^18^F-FP-DTBZ) is a promising agent with a long half-life and high binding affinity to VMAT2 (Kung et al., 2007; Hefti et al., 2010; Kilbourn and Koeppe, 2019). In addition, the new hybrid PET/MRI has been developed to study neurologic diseases (Miller-Thomas and Benzinger, 2017), which provides molecular information of the neurodegenerative pathophysiology using PET and high soft-tissue contrast and excellent spatial resolution using MRI during one scan.

Previous studies have shown that ^18^F-FP-DTBZ PET could differentiate patients with PD from healthy controls (HC) and patients with essential tremor or atypical parkinsonism. The contralateral posterior putamen has been shown to have the greatest reduction of ^18^F-FP-DTBZ uptake in all subregions of the striatum (Okamura et al., 2010; Lin et al., 2014; Alexander et al., 2017; Xu et al., 2018; Shi et al., 2019). However, there has not been any study elucidating the diagnostic value of the standardized uptake value ratio (SUVR) in distinguishing between PD and HC. Contrary to the common approach that measured the putamen in its entirety or divided it into two parts as anterior putamen and posterior putamen, applied previously in ^18^F-FP-DTBZ PET, we postulated to segment the striatum of each side into five parts as follows: caudate, anterior dorsal putamen (ADP), anterior ventral putamen (AVP), posterior dorsal putamen (PDP), and posterior ventral putamen (PVP). Our study aimed to determine the cut-off value of the SUVR with the best diagnostic power using ^18^F-FP-DTBZ PET/MR, and then verify the accuracy of the diagnostic SUVR in patients with PD and HC using routine ^18^F-FP-DTBZ PET/CT.

## Materials and methods

### Participants

Sixty-five participants, 34 patients with PD and 31 HC, were enrolled in the PET/MR derivation cohort from 2019 to 2021. In addition, 89 patients with PD and 18 HCs were enrolled in the PET/CT validation cohort from 2018 to 2021. All patients with PD were diagnosed and included using the MDS Clinical Diagnostic Criteria. Exclusion criteria included a history of psychiatric disorders, claustrophobia, serious cardio-cerebrovascular diseases, postural hypotension, malignant tumor, severe infection, heavy trauma, alcohol or drug abuse, allergy to contrast medium, or other contraindications to MRI. The Hoehn-Yahr stage and the third part of the Unified Parkinson’s Disease Rating Scale (UPDRS-III) were used to evaluate the disease staging and severity. Cognitive function, sleep disorder, depression, and anxiety in the participants were assessed using Montreal Cognitive Assessment (MOCA), Rapid Eye Movement sleep behavior disorder questionnaire-Hong Kong (RBDQ-HK), 24-item Hamilton Depression Rating Scale (HAMD-24), and Hamilton Anxiety Scale (HAMA), respectively.

All assessments and PET scanning were completed in the drug-off state, during which antiparkinsonian medication was withheld for at least 12 h. This study was approved by the Ethics Committee of Xuanwu Hospital. All participants signed a written informed consent after complete explanation of the study procedures was performed.

### Image acquisition

^18^F-FP-DTBZ was prepared and synthesized as previously reported (Lin et al., 2010; Tsao et al., 2010). All participants of the derivation cohort were examined using a hybrid 3.0-T PET/MR scanner (uPMR790, United Imaging Healthcare, Shanghai, China) with a 24-channel head/neck coil. Participants were injected int ravenously with approximately 222 MBq (6 mCi) of ^18^F-FP-DTBZ and had a quiet rest for 90 min. To reduce head movements during the scan, the participant’s head was restrained with custom-made foam pads. Acquisition parameters were as follows: field of view = 300 mm; voxel size = 1.82 mm × 1.82 mm × 2.78 mm. PET image reconstruction was performed by ordered subsets expectation maximization (OSEM) with 8 iterations, 32 subsets, and a 3.0 mm full width at half maximum (FWHM) Gaussian filter. 3D T1 BRAVO, T2, and T2-FLAIR MR images were obtained as well and used for attenuation correction and localization.

The PET/CT validation cohort was studied using a uMI 510 PET/CT scanner (United Imaging Healthcare, Shanghai, China), following a similar imaging acquisition process as mentioned above. PET images were reconstructed using time-of-flight and OSEM with 2 iterations, 24 subsets, and a 3.0 mm FWHM Gaussian filter. CT scanning parameters were as follows:120 kV, 180 mAs, pitch 0.9375.

### Image analysis

All the image data were processed and analyzed using the PMOD software (version 4.105). For the PET/MR datasets, each PET image was initially co-registered to the corresponding MR image of the same subject, and the MR image was spatially normalized to the Montreal Neurologic Institute (MNI) MR imaging template. The normalization parameters were then applied to the PET images to obtain the final spatially normalized PET image in the MNI space. A ^18^F-FP-DTBZ PET image template was created in the MNI space from the normalized PET images of the PET/CT cohort. For the PET/CT datasets, PET images were spatially normalized to the ^18^F-FP-DTBZ PET template. The volumes of interest (VOI) were defined from an atlas in the MNI space, where the striatum was bilaterally subdivided into caudate nuclei, ADP, AVP, PDP, and PVP to explore the characteristics of VMAT2 imaging in PD and HC participants. VMAT2 SUVR in target brain regions was calculated relative to the occipital cortex reference region. The contralateral VOI were taken as the regions in the opposite side to the onset of symptoms in PD subjects.

### Statistical analysis

All the analyses were conducted using Statistical Package for the Social Sciences (SPSS) software (version 26.0). Categorical and continuous data was expressed as numbers or means ± standard deviations (SD), respectively. The regional SUVRs of the ^18^F-FP-DTBZ PET images were compared using the independent *T*-test, non-parametric Mann–Whitney test, and one-way analysis of variance for group comparison between HCs and patients with PD. The Wilcoxon signed-rank test served to compare the difference in bilateral brain regions among patients with PD. The diagnostic ability and the best cut-off values of each subregion were elucidated by the receiver-operating characteristic (ROC) curves. The diagnostic accuracy, sensitivity, specificity, positive predictive rates (PPR), and negative predictive rates (NPR) of the best cut-off values were evaluated using the chi-square test. Striatal asymmetry index (SAI) was used to identify the discrepancy of bilateral SUVR in the striatum, where SAI was calculated as | (L-R)/(L + R) | × 2 × 100, with L(left) and R(right) referring to the left and right striatal SUVRs, respectively. Statistical significance was defined as a *p*-value < 0.05.

## Results

### Demographic and clinical features

Table 1 summarizes the demographic and clinical features of the participants. Regarding age and gender distribution, there was no significant difference between the HC (mean age 57.45 ± 10.52 years, 16 female, 15 male) and patients with PD (mean age 58.29 ± 13.90, 16 females, 18 male) in the derivation cohort.

**TABLE 1:** Clinical and demographic data of the PET/MR cohort.

	Age (y)	Gender	Disease Duration (m)	H-Y Scale	UPDRS III	MoCA	HAMA	HAMD-24	RBDQ-HK
HC (*N* = 31)	57.45 ± 10.52	16F/15 M	–	–	0.42 ± 1.31	25.10 ± 3.76	3.00 ± 4.86	1.93 ± 3.12	4.30 ± 5.68
	(36–71)				(0–4)	(14–30)	(0–25)	(0–14)	(0–25)
PD (*N* = 34)	58.29 ± 13.90	16F/18 M	39.47 ± 40.69	1.77 ± 0.70	22.00 ± 11.75	26.65 ± 3.07	7.43 ± 8.14	6.73 ± 10.73	14.13 ± 14.25
	(26–86)		(6–171)	(1.0–4.0)	(7–55)	(16–30)	(0–39)	(0–58)	(0–53)
*P*	0.41	0.84	–	–	<0.05	0.20	<0.05	<0.05	<0.05

H-Y Scale, Hoehn-Yahr scale; UPDRS III, motor score of unified Parkinson’s Disease rating scale; MoCA, Montreal cognitive assessment; HAMA, hamilton anxiety scale; HAMD-24, 24-item hamilton depression rating scale; RBDQ-HK, rapid eye movement sleep behavior disorder questionnaire-Hong Kong. Data are presented as mean ± SD, with ranges in parentheses.

Patients with PD had significantly higher scores of UPDRS III, HAMA, HAMD-24, and RBDQ-HK compared to HC participants, while the cognitive status did not differ between PD patients and HC based on the MOCA score.

**FIGURE 1:**
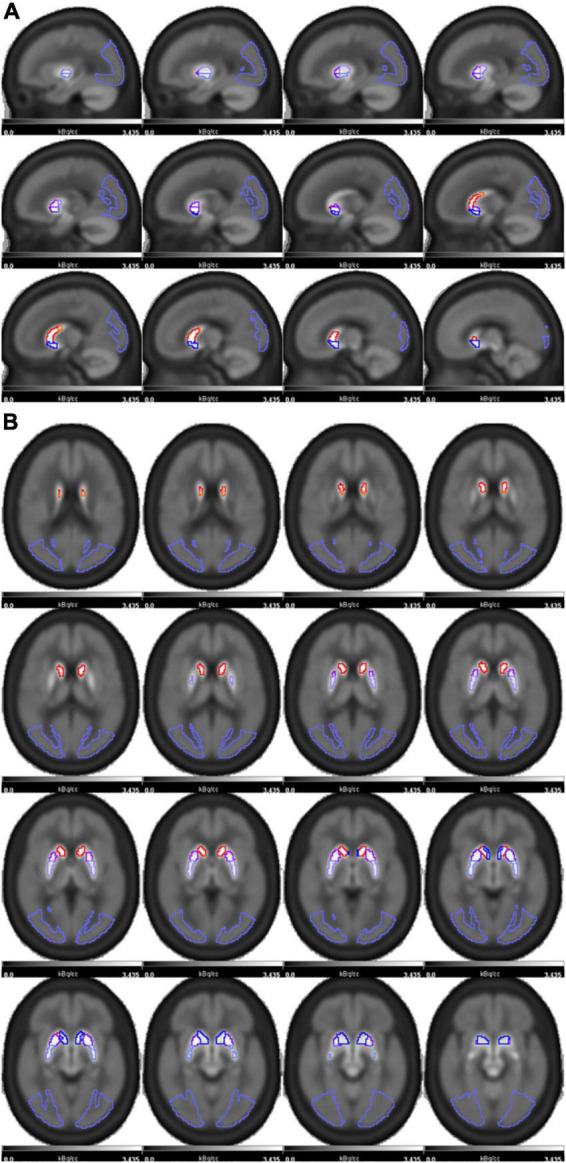
The ^18^F-FP-DTBZ PET image template with striatum regions was superimposed and shown in sagittal **(A)** and axial **(B)** positions. The striatum of each side was divided into five subregions as follows: caudate(red), anterior dorsal putamen (ADP, purple), anterior ventral putamen (AVP, dark purple), posterior dorsal putamen (PDP, pale purple), posterior ventral putamen (PVP, blue). The occipital lobe(violet) was used as the reference region.

The ^18^F-FP-DTBZ PET image template with the striatal subregions and the occipital reference regions superimposed is presented in Figure 1. Supplementary Figure 2 showed the image results of healthy control(A) and PD patients in H-Y stage 1.0 (B), 2.0 (C) of the PET/MR cohort.

The regional SUVRs of ^18^F-FP-DTBZ PET/MR between PD patients and HC were presented in Table 2. The uptake of ^18^F-FP-DTBZ in PDs was declined asymmetrically, in which contralateral regions experienced a more significant reduction. Compared with the caudate, the SUVRs reduction of the putamen was more significant. Furthermore, the uptake reduction in PD patients followed patterns such as dorsal-to-ventral and caudal-to rostral, with a more noticeably decline in the contralateral PDP (−66.4%, *P* < 0.05).

**TABLE 2:** Comparison of striatal subregional SUVRs in the PET/MR cohort.

Region	HC	I*-PD	C*-PD	*P* ^†^
Caudate	4.27 ± 0.56	3.36 ± 0.72 (−21.3%)	2.97 ± 0.70 (−30.4%)	<0.05
ADP	4.71 ± 0.58	2.65 ± 1.02 (−43.7%)	2.09 ± 0.85 (−55.6%)	<0.05
AVP	4.21 ± 0.49	2.86 ± 0.85 (−32.1%)	2.47 ± 0.77 (−41.3%)	<0.05
PDP	5.30 ± 0.71	2.38 ± 1.08 (−55.1%)	1.78 ± 0.91 (−66.4%)	<0.05
PVP	4.42 ± 0.66	2.45 ± 0.83 (−44.6%)	1.99 ± 0.62 (−55.0%)	<0.05

I*-(ipsilateral), brain regions located at the side of clinical symptoms onset (PD group); C*-(contralateral), brain regions located opposite to the onset side of clinical symptoms (PD group); ADP, anterior dorsal putamen; AVP, anterior ventral putamen; PDP, posterior dorsal putamen; PVP, posterior ventral putamen. ^†^P-value from the comparison of the SUVR of healthy controls, I*-PD and C*-PD groups.

The ^18^F-FP-DTBZ SUVRs of the ipsilateral PVP and PDP negatively correlated with disease duration, H-Y Scale, and UPDRS III scores using partial correlation analysis, after controlling for age and gender. The contralateral subregions were not correlated with disease duration, H-Y Scale, and UPDRS III (Table 3).

**TABLE 3:** Partial correlation analysis between disease duration (m), H-Y scale, UPDRS III, and regional SUVR.

	I*-Cau	I*-ADP	I*-AVP	I*-PDP	I*-PVP	C*-Cau	C*-ADP	C*-AVP	C*-PDP	C*-PVP
Disease Duration (m)	*P* = 0.106	***r* = −0.399** ***P* < 0.05**	*P* = 0.055	***r* = −0.372** ***P* < 0.05**	***r* = −0.385** ***P* < 0.05**	*P* = 0.215	*P* = 0.136	*P* = 0.173	*P* = 0.253	*P* = 0.136
H-Y Scale	*P* = 0.342	*P* = 0.093	*P* = 0.169	***r* = −0.369** ***P* < 0.05**	***r* = −0.365** ***P* < 0.05**	*P* = 0.574	*P* = 0.425	*P* = 0.561	*P* = 0.521	*P* = 0.342
UPDRS III	***r* = −0.416** ***P* < 0.05**	***r* = −0.365** ***P* < 0.05**	*P* = 0.132	***r* = −0.392** ***P* < 0.05**	***r* = −0.386** ***P* < 0.05**	*P* = 0.081	*P* = 0.158	*P* = 0.431	*P* = 0.319	*P* = 0.196

H-Y Scale, Hoehn-Yahr scale; UPDRS III, motor score of unified Parkinson’s Disease rating scale; I*-(ipsilateral), brain regions located at the side of clinical symptoms onset (PD group); C*-(contralateral), brain regions located opposite to the side of clinical symptoms onset (PD group); Cau, Caudate; ADP, anterior dorsal putamen; AVP, anterior ventral putamen; PDP, posterior dorsal putamen; PVP, posterior ventral putamen. P, p-value; r, correlation coefficient. We conducted this partial correlation analysis after controlling the interference of age and gender in the PET/MR cohort. P < 0.05 with correlation coefficient are shown in bold.

### Diagnostic performance of the cut-off value in the PET/MR cohort

The SAI was higher in patients with PD compared to HC (4.07 ± 3.00 vs. 19.06 ± 12.06, *P* < 0.05) in the derivation cohort. Supplementary Figure 1A showed that the best cut-off value of SAI was 11.22 (Accuracy of SAI: Area under the ROC curve = 0.904; 95% CI: 0.832–0.976; *P* < 0.05), and the ROC curve analysis showed that the SAI had a sensitivity of 100% and specificity of 100% in differentiating PD from HC, which also indicated that the SUVR of the bilateral caudate and putamen of patients with PD had significant differences.

The area under the ROC curve of caudate, ADP, AVP, PDP, and PVP in the contralateral side were 0.935 (95% CI: 0.874–0.997), 0.973 (0.919–1.000); 0.972 (0.917–1.000); 0.973 (0.919–1.000), and 0.977 (0.933–1.000), respectively. In contrast, the AUC of caudate, ADP, AVP, PDP, and PVP in the ipsilateral side were 0.854 (0.760–0.948); 0.954 (0.893–1.000); 0.922 (0.844–1.000); 0.970 (0.914–1.000), and 0.958 (0.900–1.000), which is lower than the AUC of the corresponding subregion in the contralateral side. Supplementary Figure 1B showed the sensitivity, specificity, and AUC of the ROC curve analysis of the regional diagnostic SUVRs in differentiating PD from HC, in which we found that the contralateral PDP and PVP subregions had a better diagnostic performance with the same sensitivity, specificity, positive predictive value (PPV), and negative predictive value (NPV) as 97.1% (95% CI: 82.9–99.8%), 100% (95% CI:86.3–100%), 100% (95% CI: 87.0–100%), 96.9% (95% CI: 82.0–99.8%), respectively.

### Validation of the cut-off value in the PET/CT cohort

We performed blind validation in the ^18^F-FP-DTBZ PET/CT cohort, which had 18 HCs (mean age 65.28 ± 10.93, 7 females, 11 male) and 89 patients with PD (mean age 53.59 ± 13.39, 39 females, 50 male). The mean disease duration of PDs in the validation cohort were 54.65 ± 64.80 (range: 2.7–360.3) months. The H-Y Scale of PDs in this cohort were 2.08 ± 0.68 (1.0–4.0). Furthermore, the UPDRS III score of HC and PDs were significantly different, which were 0.83 ± 1.47 (0–5) and 31.89 ± 16.77 (7–84), respectively.

**FIGURE 2:**
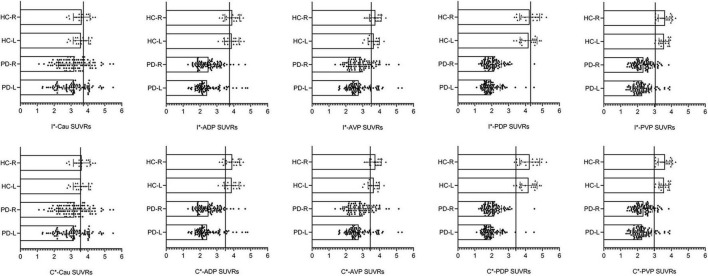
Scatterplots with bars of the diagnostic SUVR of each subdivisions. HC-R, the right side of healthy controls in the validation cohort; HC-L, the left side of healthy controls in the validation cohort; PD-R, the right side of PD patients in the validation cohort; PD-L, the left side of PD patients in the validation cohort; I*-(ipsilateral), brain regions located at the side of clinical symptoms onset (PD group) in the derivation cohort; C*-(contralateral), brain regions located opposite to the side of clinical symptoms onset (PD group) in the derivation cohort; Cau, caudate; ADP, anterior dorsal putamen; AVP, anterior ventral putamen; PDP, posterior dorsal putamen; PVP, posterior ventral putamen. We presented the right and left side of healthy controls and PD patients in the validation cohort, but did not distinguish between the ipsilateral and contralateral sides of the PD patients. Then, the optimal diagnostic SUVRs of each subregion were put into the corresponding subregion of the validation cohort, and those with less than the corresponding diagnostic SUVR were defined as “PD patients.” Scatterplots with bars of each subdivision showed that the diagnostic SUVR of contralateral posterior dorsal putamen had the best diagnostic accuracy of 98.1%. The vertical line represents the cut-off value defined in the PET/MRI cohort.

If any subregional SUVR in the PET/CT cohort was less than the corresponding best cut-off value in the PET/MR cohort, we defined these participants as patients with PD, and others were defined as HC blindly. Figure 2 demonstrated that the diagnostic cut-off value of the contralateral PDP (3.43) had the best diagnostic ability and consistency.

The diagnostic consistency between clinical diagnosis and imaging diagnosis was confirmed with Kappa test (Kappa = 0.933, *P* < 0.05) in the validation cohort, which showed a sensitivity of 98.9% (95% CI: 93.0–99.9%), specificity of 94.4% (95% CI: 70.6–99.7%), positive likelihood ratio 98.9% (95% CI: 93.0–99.9%), negative likelihood ratio 94.4% (95% CI: 70.6–99.7%), and an accuracy of 98.1%, respectively.

## Discussion

This is the first report of the diagnostic cut-off value for PD based on the SUVR of ^18^F-FP-DTBZ PET/MR. After the blind validation in an independent cohort, we found that the SUVR of the contralateral PDP contributed most to distinguishing patients with PD from HC with sensitivity, specificity, PPV, NPV, and diagnostic accuracy of 98.9, 94.4, 98.9, 94.4, and 98.1%, respectively. Kappa-coefficient of 0.933 (*P* < 0.05) demonstrated a high test-to-retest reliability between clinical diagnosis and molecular imaging diagnosis. This *in vivo* ^18^F-FP-DTBZ PET imaging study also showed the uneven pattern of dopaminergic neurodegeneration (caudal > rostral, dorsal > ventral), consistent with pathologic studies (Kish et al., 1988). Besides, the current study found negative correlations between ^18^F-FP-DTBZ uptake of the ipsilateral PDP and PVP, and disease duration, H-Y Scale, and UPDRS III after adjusting for age and gender, suggesting that ^18^F-FP-DTBZ PET may be a biomarker for monitoring the progression of PD. The contralateral putamen showed no correlation with disease duration, H-Y Scale, and UPDRS III score, probably due to the limited sample size or a floor effect resulting from the fact that the contralateral putamen was damaged more severely (Okamura et al., 2010).

With the main neuropathological foundation of the loss of dopaminergic neurons in the ventrolateral SN (Blesa et al., 2022), PD is currently diagnosed based on clinical manifestations and drug responsiveness, requiring detailed history and neurologic examination to rule out other atypical parkinsonian disorders (Homayoun, 2018). Early and accurate identification of PD is very important, as it facilitates the progress of future disease-modifying therapies, avoids unnecessary examinations and treatments, and reduces costs and side effects. However, this goal is still to be achieved. For movement disorders specialists, clinical diagnostic accuracy was 79.6% at the initial assessment, which increased to 83.9% after follow-up (Rizzo et al., 2016) in PD patients. The overall efficiency of clinical diagnosis in PD is not satisfactory. A clinicopathologic study indicated only 53% of clinical diagnostic accuracy in early medication-responsive PD (<5 years’ duration), and 85% in advanced PD responsive to medication (Adler et al., 2014). In contrast to neuropathological biopsy, functional imaging is non-invasive for investigating the synthesis and metabolism of the dopaminergic system at a molecular level *in vivo* (Arena and Stoessl, 2016). Neuroimaging biomarkers are urgently needed to improve clinical diagnostic accuracy and monitor disease progression of PD *in vivo*.

Currently, the main radiotracers used for imaging abnormalities of the presynaptic dopaminergic system based on PET includes ^11^C-RTI-32, ^11^C-Nomifensine, ^11^C-Methylphenidate, ^11^C-PE2I, ^18^18F-CFT, ^18^F-FP-CIT (DAT activity), ^11^C-DTBZ, ^18^F-FP-DTBZ (VMAT2 activity), and ^18^F-DOPA (AADC activity). Furthermore, the tracers based on Single Photon Emission Computed Tomography (SPECT) includes ^9^9Tc-TRODAT-1, ^123^I-β-CIT, ^123^I-FP-CIT, ^123^I-PE2I, and ^123^I-IPT (DAT activity) (de Natale et al., 2018). Although DAT and VMAT2 are critical for DATs localized in the presynaptic dopaminergic neurons, DAT is more susceptible to aging and gender in contrast to VMAT2 and AADC (Richardson et al., 2008; Nobili et al., 2013). Haycock et al. (2003) measuring AADC, VMAT2, and DAT levels in post-mortem human striatum have shown that the level of VMAT2 (unlike AADC and DAT) was relatively constant with age in adulthood. Both *in vivo* (Chan et al., 1999; Troiano et al., 2010; Lin et al., 2013) and *in vitro* PET studies (Haycock et al., 2003; Tong et al., 2011) illustrated no significant aging-related decline of VMAT2 density in the nigrostriatal system, indicating that this target protein was relatively stable. Thus, age may not be a crucial confounding factor of ^18^F-FP-DTBZ PET image (Lin et al., 2013), and in this study we calculated the diagnostic SUVR without considering aging. Age stratification and diagnostic values of different age groups may be done with larger sample size in the future.

To date, ^123^I-FP-CIT SPECT (DaTSCAN) is the only radiotracer approved for the diagnosis of suspected parkinsonian syndromes, especially for PD, by the European Medicines Agency in 2000 and the U. S. Food and Drug Administration in 2011. Remarkably, while the research value of ^123^I-FP-CIT images and ^123^I-FP-CIT SPECT seems undoubted, the controversy has long been about its clinical utility (Fuente-Fernández R.d.l, 2012a,b; Fuente-Fernández R.d.l Lövblad, 2014; Erro et al., 2016; Hickey et al., 2017). The results of ^123^I-FP-CIT SPECT were usually derived from visual reading manually or computer-aided semiquantitative analysis. Nevertheless, visual reading is time-consuming, labor-intensive, and prone to human bias. In contrast, our study used a brain atlas to automatically outline the VOIs to minimize human bias. Despite the high specificity of semiquantitative analysis, it is not generally used in clinical practice for ^123^I-FP-CIT SPECT. It is worth noting that a discrepancy sometimes exists between visual ratings and semiquantitative analysis in the form of normal visual readings linked with abnormal semiquantitative analysis or abnormal visual readings linked with normal semiquantitative analysis. Inevitably, this will lead to increasing numbers of false-positive and false-negative cases (Walker et al., 2007; Fuente-Fernández R.d.l, 2012b). Furthermore, approximately 10–20% of patients meeting the PD diagnostic criteria clinically, who accomplished dopaminergic imaging in neuroprotective trials of PD, had “scans without evidence of dopaminergic deficit (SWEDD)” (Benamer et al., 2003; Whone et al., 2003; Parkinson Study Group Precept Investigators, 2007). This group of cases showed high heterogeneity, and several clinical futures masqueraded as PD in many cases. Some patients with SWEDD may support an alternative diagnosis as adult-onset dystonia or other forms of tremor (Schneider et al., 2007; Bain, 2009; Schwingenschuh et al., 2010), whereas part of them still had PD as the most likely clinical diagnosis. Thus, the discordance between definite clinical manifestation and unexpected normal DAT imaging led researchers to re-diagnose the disease through detailed clinical reassessment and diagnostic work-up. Several prospective follow-up studies using both visual rating and semiquantitative analysis of DAT imaging have demonstrated that some (perhaps most) of these SWEDDs cases may actually have presynaptic nigrostriatal degeneration (Marshall et al., 2006; Sixel-Döring et al., 2011; Menéndez-González et al., 2014). Therefore, this supports our speculation that the initial normal dopaminergic imaging is less sensitive to detect this change in the early PD. To prove this, Hickey et al. (2017) enrolled 20 participants with probable *de novo* PD in a prospective comparative pilot study, in which ^123^I-FP-CIT SPECT was found to have no additional impact to clinical diagnosis. Taken together, irrespective of the SWEDD phenomenon or the inconsistency between visual ratings and quantitative analyses, ^123^I-FP-CIT SPECT has a tendency for misdiagnosis in the early stage of PD with relatively low sensitivity and a high false-negative rate. Given that SPECT imaging has a low resolution compared to PET imaging, the clinical utility of ^123^I-FP-CIT SPECT will be further limited, which also encouraged the development of the ^18^F labeled FP-CIT radiotracer. This tracer is competitive with a longer half-life and fewer radioactive metabolites (Chaly et al., 1996). In contrast to ^123^I-FP-CIT SPECT, ^18^F-FP-CIT PET has improved image resolution and clinical workflow including shorter injection-to-scan and acquisition times. Nonetheless, FP-CIT (either ^18^F- or ^123^I- labeled) targets basically on DAT and serotonin transporter (SERT) (Scheffel et al., 1997; Booij et al., 2007), which are expressed on the surface of terminals and are vulnerable to influencing factors. The uptake of ^18^F-FP-CIT could be impacted by DA-depleting drugs (Kilbourn et al., 1992; Gordon et al., 1996), dopaminergic medications (Borght et al., 1995b), and selective serotonin reuptake inhibitors (SSRIs) (Booij et al., 2007; Pontone et al., 2009, 2013). On the contrary, the ^18^F-FP-DTBZ binds to VMAT2 which is expressed inside the terminal and the uptake of ^18^F-FP-DTBZ is less impacted by drugs or compensation effects. Several animal experimental studies indicated that VMAT2 binding was less prone to dopaminergic medications compared with DAT or AADC activity (Borght et al., 1995b; Kilbourn et al., 1996; Frey et al., 1997; Fleckenstein et al., 2000; Kemmerer et al., 2003). However, because of the possible downregulation of DAT in the early phase of PD, the FP-CIT might be more sensitive to the early detection of dopaminergic disruption. For the radiopharmaceutical part, it should be noted that total the synthesis time of ^18^F-FP-CIT PET was 80 min (Chaly et al., 1996; Lee et al., 2007), which is twice than that of ^18^F-FP-DTBZ (approximately 40 min) (Zhu et al., 2010). A quantitative autoradiography study showed that the density or imaging resolution of DAT was significantly lower than that of VMAT2 in striatal regions (Sun et al., 2012), and was difficult for subregional analysis. Taken together, the ligands targeting VAMT2 have been proven to be less vulnerable to dopaminergic drugs, age, and compensatory effects comparing to the tracers targeting DAT, and the VMAT2 tracer ^18^F-FP-DTBZ, with a relatively long half-life (110 min), is more widely used compared to ^11^C-DTBZ in clinical settings (Kung et al., 2007; Hefti et al., 2010; Kilbourn and Koeppe, 2019). However, additional studies across different centers are required to further evaluate the role played by different presynaptic dopaminergic tracers.

Asymmetry degeneration of dopaminergic neurons and subsequent clinical lateralization of motor symptoms are a unique hallmark of PD, which had been evidenced by neuropathological studies compared to atypical parkinsonian syndromes (Riederer et al., 2018), such as multiple system atrophy, Lewy body dementia, and progressive supranuclear palsy. In the current study, it is clear that the SAI of patients with PD was significantly higher than that of HC, indicating significant clinical lateralization in patients with PD. Nonetheless, the ROC curve in our study showed that SAI had a relatively lower sensitivity of 70.59% in differentiating PDs from HC, which was not satisfactory.

Our results first used ^18^F-FP-DTBZ PET/MR to obtain the best diagnostic SUVR, and then validated it blindly in another ^18^F-FP-DTBZ PET/CT cohort, which demonstrated a high sensitivity, specificity, and accuracy, although further neuropathology validation is still indispensable. However, a larger sample size of the derivation and validation cohorts in the future is needed to determine the diagnostic SUVR for generally clinical utility. Another limitation of this study is that we did not stratify the patients with PDs and HCs based on age. For example, every 10 years as a layer to achieve the diagnostic SUVR of different age groups, so that the effect of the degree of dopaminergic neurodegeneration due to aging may be reduced.

## Conclusion

Our study provided an intuitive index of the diagnostic SVUR *in vivo* of ^18^F-FP-DTBZ PET/MRI, which may be a potential diagnostic tool in differentiating PD from normal individuals and monitoring the disease progression. The SUVR of ^18^F-FP-DTBZ declined notably in the ipsilateral striatum, suggesting that ^18^F-FP-DTBZ PET may be a sensitive biomarker in detecting dopaminergic neurodegeneration in the premotor/early stage of PDs.

## Data availability statement

The original contributions presented in this study are included in the article/Supplementary Material, further inquiries can be directed to the corresponding author.

## Ethics statement

The studies involving human participants were reviewed and approved by the Ethics Committee of Xuanwu Hospital. The patients/participants provided their written informed consent to participate in this study. Written informed consent was obtained from the individual(s) for the publication of any potentially identifiable images or data included in this article.

## Author contributions

S-YL and PC: study concept, design, and manuscript revision for important intellectual content. S-YL and X-LL: clinical data collection. S-YL and OB: imaging data analysis. H-WQ, T-BS, GT, and JL: imaging data collection. X-LL: manuscript writing. All authors read and approved the final version of the manuscript for submission.

## Conflict of interest

The authors declare that the research was conducted in the absence of any commercial or financial relationships that could be construed as a potential conflict of interest.

## Publisher’s note

All claims expressed in this article are solely those of the authors and do not necessarily represent those of their affiliated organizations, or those of the publisher, the editors and the reviewers. Any product that may be evaluated in this article, or claim that may be made by its manufacturer, is not guaranteed or endorsed by the publisher.
