# Corrigendum: Diagnostic value of striatal ^18^F-FP-DTBZ PET in Parkinson's disease

**DOI:** 10.3389/fnagi.2022.1029024

**Published:** 2022-09-14

**Authors:** Xiu-Lin Liu, Shu-Ying Liu, Olivier Barret, Gilles D. Tamagnan, Hong-Wen Qiao, Tian-Bin Song, Jie Lu, Piu Chan

**Affiliations:** ^1^Department of Neurology, Xuanwu Hospital, Capital Medical University, Beijing, China; ^2^Chinese Institute for Brain Research (CIBR), Beijing, China; ^3^CEA, CNRS, MIRCen, Laboratoire des Maladies Neurodégénératives, Université Paris-Saclay, Fontenay-aux-Roses, France; ^4^Mental Health PET Radioligand Development (MHPRD) Program, Yale University, New Haven, CT, United States; ^5^Department of Radiology and Nuclear Medicine, Xuanwu Hospital, Capital Medical University, Beijing, China; ^6^National Clinical Research Center for Geriatric Diseases, Beijing, China

**Keywords:** ^18^F-FP-DTBZ, VMAT2, Parkinson's disease, diagnostic value, positron emission tomography

In the published article, an author name was incorrectly written as “Tian-Bing Song.” The correct spelling is “Tian-Bin Song.”

The authors apologize for this error and state that this does not change the scientific conclusions of the article in any way. The original article has been updated.

## Publisher's note

All claims expressed in this article are solely those of the authors and do not necessarily represent those of their affiliated organizations, or those of the publisher, the editors and the reviewers. Any product that may be evaluated in this article, or claim that may be made by its manufacturer, is not guaranteed or endorsed by the publisher.

