# Supplementary material for: Diagnostic value of striatal 18F-FP-DTBZ PET in Parkinson’s disease
Source: Front Aging Neurosci. 2022 Jul 22;14:931015. doi: 10.3389/fnagi.2022.931015 (PMC9355024; doi:10.3389/fnagi.2022.931015)
Supplement: Supplementary file 1 [file Data_Sheet_1.docx]

Supplementary Material

**Table 1.** Striatal Asymmetry Index of the Participants in the PET/MRI Cohort.

| **HC SAI** | **PD SAI** | ***P*** | **the Best Cut-off Value** | **AUC** | **Sensitivity (%)** | **Specificity (%)** |
| --- | --- | --- | --- | --- | --- | --- |
| 4.07 ± 3.00 | 19.06 ± 12.06 | <0.05 | 11.22 | 0.904  (0.832–0.976) | 70.59  (53.83–83.17) | 100  (88.97–100.00) |

Abbreviations: SAI = Striatal Asymmetry Index; AUC = Area Under the ROC Curve.

**Table 2.** The best cut-off value of SUVRs in Defined Regions of PDs and HCs

| **Region** | **Best**  **Cut-off Value** | **AUC** | **Sensitivity**  **(%)** | **Specificity (%)** | **PPV** | **NPV** |
| --- | --- | --- | --- | --- | --- | --- |
| **I^*-^Cau** | 3.73 | 0.854  (0.760–0.948) | 73.5  (55.3–86.5) | 90.3  (73.1–97.5) | 89.3  (70.6–97.2) | 75.7  (58.4–87.6) |
| **I-ADP** | 3.74 | 0.954  (0.893–1.000) | 88.2  (71.6–96.2) | 100  (86.3–100) | 100  (85.9–100) | 88.6  (72.3–96.3) |
| **I-AVP** | 3.50 | 0.922  (0.844–1.000) | 88.2  (71.6–96.2) | 100  (86.3–100) | 100  (85.7–100) | 88.6  (72.3–96.3) |
| **I-PDP** | 4.30 | 0.970  (0.914–1.000) | 97.1  (82.9–99.8) | 96.8  (81.5–99.8) | 97.1  (82.9–99.8) | 96.8  (81.5–99.8) |
| **I–PVP** | 3.04 | 0.958  (0.900–1.000) | 88.2  (71.6–96.2) | 100  (86.3–100) | 100  (85.9–100) | 88.6  (72.3–96.3) |
| **C^*^-Cau** | 3.56 | 0.935  (0.874–0.997) | 85.3  (68.2–94.5) | 96.8  (81.5–99.8) | 96.7  (80.9–99.8) | 85.7  (69.0–94.6) |
| **C-ADP** | 3.50 | 0.973  (0.919–1.000) | 97.1  (82.9–99.8) | 100  (86.3–100) | 100  (87.0–100) | 96.9  (82.0–99.8) |
| **C-AVP** | 3.44 | 0.972  (0.917–1.000) | 97.1  (82.9–99.8) | 100  (86.3–100) | 100  (87.0–100) | 96.9  (82.0–99.8) |
| **C-PDP** | 3.43 | 0.973  (0.919–1.000) | 97.1  (82.9–99.8) | 100  (86.3–100) | 100  (87.0–100) | 96.9  (82.0–99.8) |
| **C-PVP** | 2.98 | 0.977  (0.933–1.000) | 97.1  (82.9–99.8) | 100  (86.3–100) | 100  (87.0–100) | 96.9  (82.0–99.8) |

Abbreviations: I^*^–(ipsilateral) = brain regions located at the onset side of clinical symptoms (PD group); C^*^–(contralateral) = brain regions located opposite to the onset side of clinical symptoms (PD group); Cau = Caudate; ADP = Anterior Dorsal Putamen; AVP = Anterior Dorsal Putamen; PDP = Posterior Dorsal Putamen; PVP = Posterior Ventral Putamen; PPV = positive predictive value; NPV = negative predictive value; AUC = Area Under the ROC Curve.


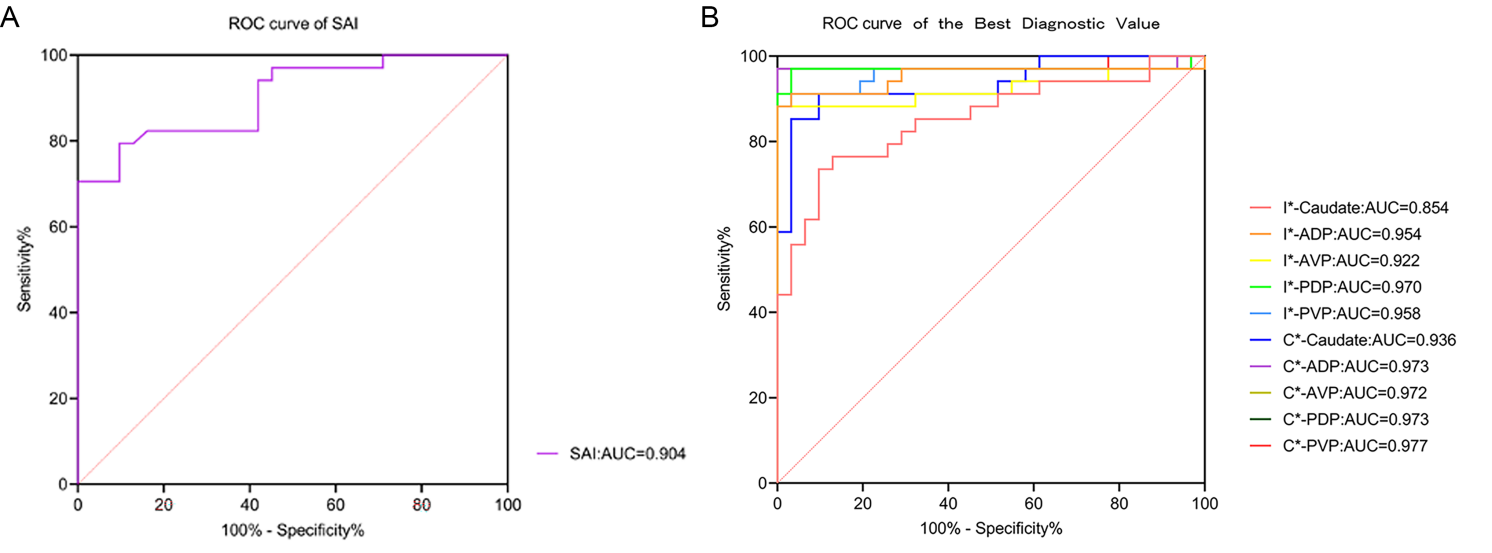


**Figure 1.** ROC curve analysis of SAI and regional SUVRs in differentiating PD from HC. The diagnostic performance of SAI is not satisfactory.

Abbreviations: I^*^-(ipsilateral) = brain regions located at the side of clinical symptoms onset (PD group); C^*^-(contralateral) = brain regions located opposite to the side of clinical symptoms onset (PD group); ADP = anterior dorsal putamen; AVP = anterior ventral putamen; PDP = posterior dorsal putamen; PVP = posterior ventral putamen.


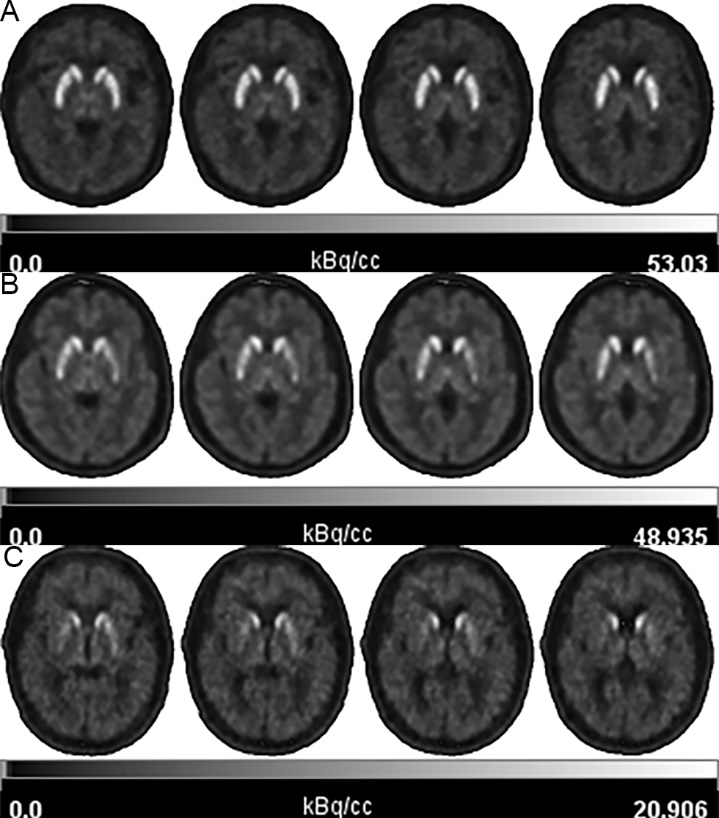


**Figure 2.** Image results of healthy control(A) and PD patients in H-Y stage 1.0(B), 2.0(C).
